# Bilateral Facial Palsy in Rapidly Progressive Course of Wegener's Granulomatosis: A Case Report

**DOI:** 10.1155/2013/875108

**Published:** 2013-09-25

**Authors:** Anna Roszkowska, Monika Morawska-Kochman, Hanna Temporale, Małgorzata Sikorska-Żuk, Tomasz Kręcicki

**Affiliations:** Department of Otolaryngology Head and Neck Surgery, Wroclaw Medical University, Borowska 213, 50-556 Wrocław, Poland

## Abstract

*Introduction*. Wegener's granulomatosis belongs to a group of systemic vasculitis diseases, which is characterized by necrotizing vasculitis and presence of granulomas. In a lot of cases, the first symptoms of the disease are observed in the head and neck region, but the bilateral facial nerve palsy occurs very rarely. *Objective*. The objective of our report was to describe the unusual course of Wegener's granulomatosis with the bilateral facial nerve paralysis, which subsided after application of steroids and immunosuppressive therapy in combination with surgical treatment. *Results and Conclusions*. Hearing loss may precede other symptoms in Wegener's granulomatosis. Ear pain and otorrhea may suggest the diagnosis of bacterial purulent otitis media and delay the proper diagnosis. In the presented case, considering the clinical course, it was necessary to apply both pharmacological and surgical treatments.

## 1. Introduction

Wegenr's granulomatosis (WG) is a rare systemic autoimmune disease. It is estimated that in Europe there are 25–150 individuals per million suffering from WG and 5–10 new cases per million annually [1]. The increased incidence is recorded in the Scandinavian countries, compared to the countries of southern Europe. Using the “Northsouth” factor, in Norway an average of 12 new cases per million inhabitants per year was recorded and in Spain four times less 3 per million [2]. The new nomenclature defines this disease as *granulomatosis with polyangiitis*—GPA [3]. This name better reflects the nature of this pathology. *granulomatosis *refers to necrotizing granulomatous vasculitis, and *polyangiitis *concerns mainly small-diameter vessels belonging to the group of diseases called *microscopic polyangiitis* MPA [4].

The disease occurs in both young and adults, with no gender predilection. The average age at diagnosis is 40 [5, 6]. Changes are located typically in both upper and lower respiratory tract and in kidneys (rapidly progressive glomerulonephritis). Depending on the number of affected organs, there are two forms of the disease, isolated form and generalized form. Any organ can be affected. Occasionally, it can be the nervous system, gastrointestinal tract, heart, eyeball, osteoarticular system, and mammary gland [1, 7]. Current treatment regimens based on combination therapy with steroids and immunosuppressive drugs (cyclophosphamide, methotrexate) allow for remission in 70–80% of cases [2, 8, 9]. Lack of treatment might be fatal in 80–82% patients in the first year after diagnosis [9, 10]. Patients with WG have an average survival of 5 months [1, 6]. 

The WG course can be variable. In 25% of cases the disease course is fulminant; it can lead to multiorgan failure and death [3]. Due to the lack of specific symptoms in the early stages, it may be poorly recognized and treated, significantly reducing the patient's prognosis. The reported case represents the rapidly progressive WG with an unusual presentation of bilateral facial nerve paralysis.

## 2. The Case Report

A 41-year-old man, white-collar worker, was urgently admitted to the Department of Otolaryngology Head and Neck Surgery of the Wroclaw University Hospital due to hearing loss and severe both sided ear pain, significantly increasing within two days. Initially bilateral acute otitis media with purulent discharge from the right ear was diagnosed. In anamnesis, there was no fever or other symptoms of acute upper respiratory tract infection. The patient complained of a hearing loss, gradually increasing for the past three weeks, with an accompanying feeling of blocked ears.

**Figure 1:**
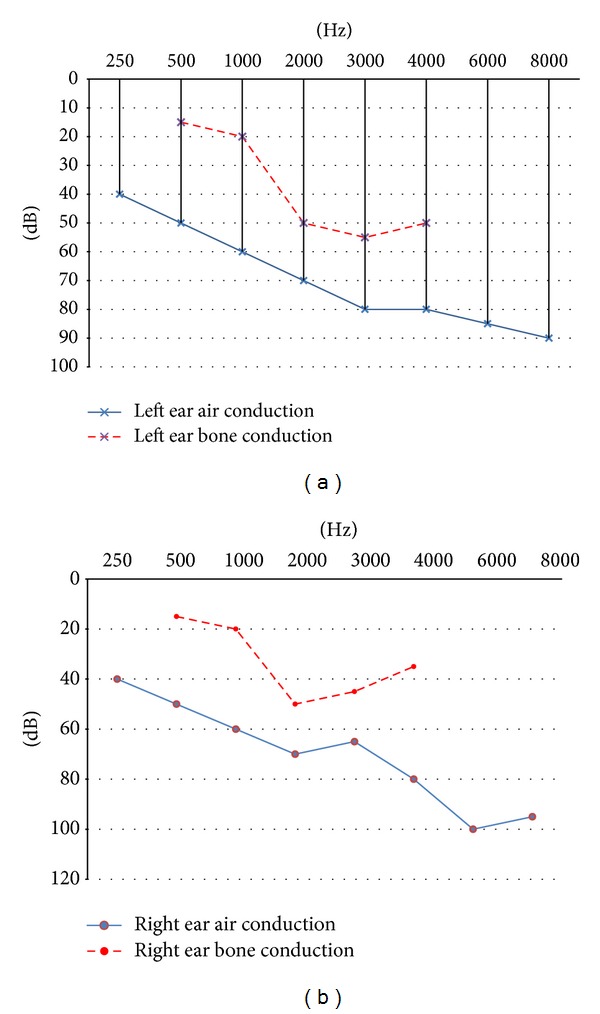
Presenting bilateral mixed hearing loss (full description in the text).

The patient's medical history was otherwise insignificant. On admission, intravenous antibiotic therapy was administered, ciprofloxacin hydrochloride (Cipronex 400 mg every 12 hours) and metronidazole (500 mg every 8 hours). Endoscopic examination was performed, in which the tympanic membrane redness on the left side and the tympanic membrane perforation with the purulent otorrhea on the right side were found. In the nasopharynx, oropharynx, and laryngopharynx, there were no pathological changes. Nasal mucosa of the left nasal cavity showed granulomatous changes with ulcerations, covered with lots of clots and drying mucopurulent secretions. No changes of the right nasal cavity were observed. Blood tests showed, WBC 11.62  thousand/uL (normal range 4–10), neutrophils 64.7% (normal range 40–70), and CRP 7.33 mg/L (normal range −5,0). Audiological tests were also performed. Pure tone audiometry revealed symmetric, severe bilateral mixed hearing loss in mid- and high-frequencies with 40 dB of air-bone gap (Figure 1).

In impedance audiometry type B curve for the left ear was obtained. Therefore, left-sided paracentesis was performed immediately. Obtained purulent secretion was sent for microbiological examination, which revealed *Staphylococcus coagulase-negative*.

**Figure 2:**
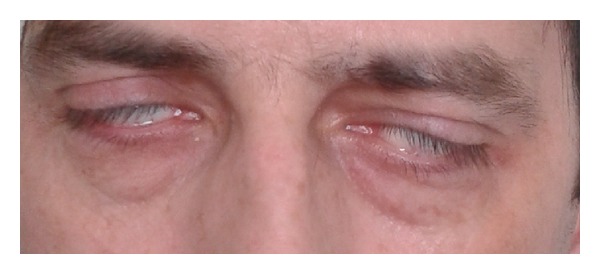
Bilateral facial nerve palsy, incompetence of palpebral fissures.

Four days later, the patient's condition deteriorated significantly. The patient reported bilateral tinnitus, right-ear pain radiating to maxilla, and severe headaches with facial numbness. Sudden bilateral peripheral facial nerve palsy occurred the following day. It was rated the fifth grade according to the House-Brackmann scale (Figure 2). No other neurological symptoms were observed. Control morphology showed neutrophils 92.4% (normal range 40–70), leukocytes 8.92 thousand/uL (normal range 4–10), and CRP 230 mg/L (normal range −5.0). WG was suspected and ANCA blood tests were collected. Marked with the ELISA-test (enzyme-linked immunosorbent assay), pANCA were negative and c-ANCAs were positive: 80.45 RU/mL. Renal function tests (urea, creatinine) were within normal range. The chest X-ray showed lung fields without focal lesions and mediastinal margins within normal limits.

**Figure 3:**
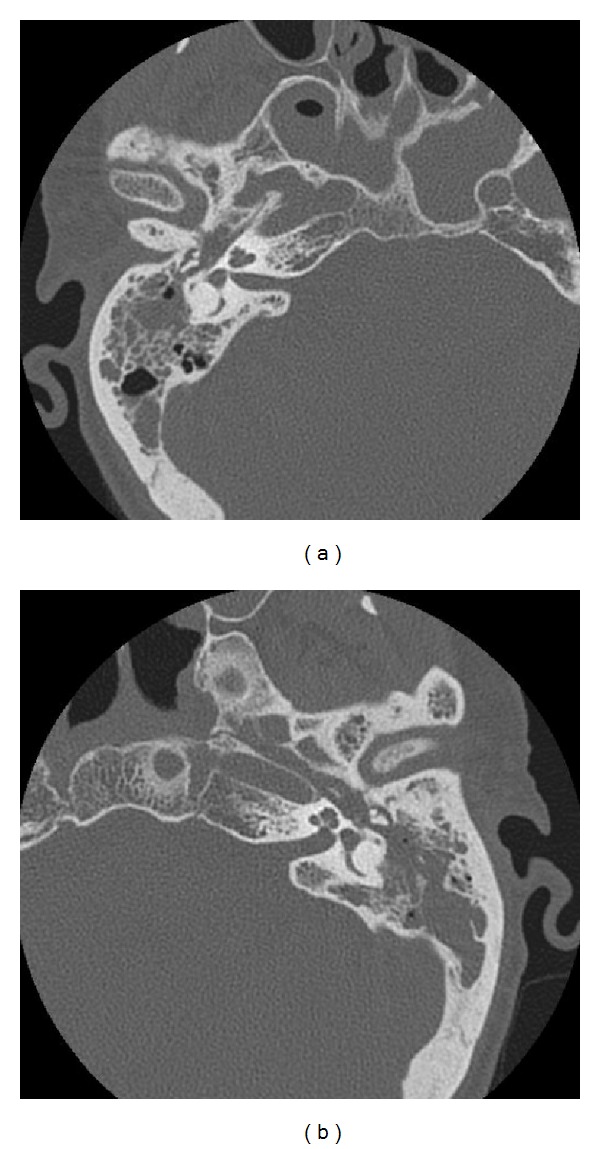
CT scan showing inflammatory tissue filling both left and right tympanic cavities.

Computed tomography scans (CT) of the temporal bones were performed in helical acquisition technique with cross layer thickness 0.625 mm and evaluated by using both soft tissue and bone window MPR reconstruction. In the right ear the cells of the mastoid process were mostly airless. The tympanic cavity was filled with soft tissue mass surrounding the ossicles, suggesting inflammatory granulation tissue. There was no evidence of dehiscence or fistulae in semicircular canals. Visible facial nerve canal had correct width in vertical section, but it could not be traced in the horizontal section. Erosion of the duct from the tympanic cavity was suspected. In the left ear almost all of the cells of the mastoid process were airless (Figure 3).

Magnetic resonance imaging (MRI) was performed in T1, T2-dependent, and T1-dependent images after intravenous contrast agent. MRI showed cerebral and intracranial cerebral fluid spaces within the normal range. The mastoid process cells were filled with fluid-inflammatory changes on both sides. The massive circular mucosal thickening in the left maxillary sinus, in the sphenoid sinus, and right front sinus was found. In addition, the sphenoid sinus fluid level was seen as an exacerbation of inflammatory process.

The patient was qualified for antromastoidectomy. Mastoid cavity and antrum were opened. They were filled with inflammatory granulation tissue. The course of the facial nerve canal was revealed. There were no signs of the facial nerve bone canal erosion. The histopathological examination of the material collected during the surgery described the extensive inflammatory granulation tissue with basophilic foci of necrosis clots. Focal acute inflammation of the arteries and veins with the fibrous necrosis of the walls was showed. Neutrophils and eosinophils inflammatory infiltration was presented. Based on histopathological, immunohistochemical, and clinical studies the diagnosis of WG was confirmed.

The patient was transferred to the Department of Rheumatology of Wroclaw University Hospital, where treatment of three Solu-Medrol pulses (3 × 500 mg), methylprednisolone (32 mg/per day), and Biseptol (960 mg every 12 hours) was administered. After two weeks of the treatment, gradual resolution of facial nerve palsy was observed. The patient reported general improvement and hearing recovery, which was confirmed in audiometric test performed one month after initiation of the treatment. The hearing improvement of 10 to 30 dB was noted, with little change in the cochlear reserve (hearing gap?). Despite the treatment, values of the inflammation parameters were still high (CRP 208 mg/L, OB 106 mm/h). The patient was under the continuous care of ENT and Rheumatology Outpatient Clinic of the Wroclaw University Hospital.

During a four-month follow-up treatment, the patient received Endoxan infusions (cyclophosphamide) in sequence 1000 mg, 800 mg, 800 mg, respectively with the protection of Uromitexan. Total dose of cyclophosphamide 2.6 g was administered. Taking into account the positive response to the treatment, the dose of methylprednisolone was reduced to 28 mg/per day, with the further dose reduction of 4 mg in two weeks to the maintenance dose of 24 mg/per day.

**Figure 4:**
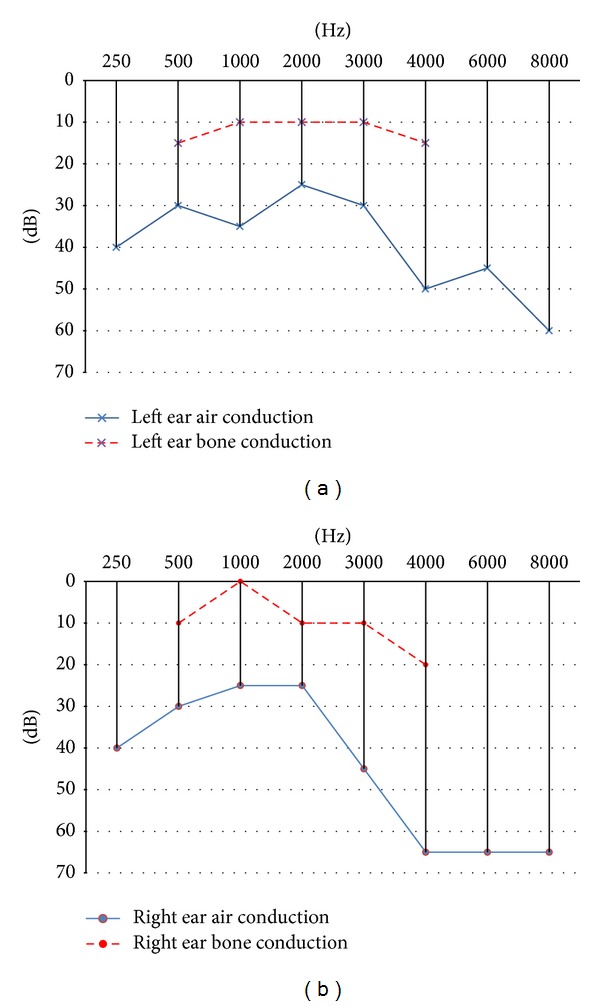
Control pure-tone audiometry, performed after four months of treatment, description in the text.

After four months, the control CT scan of temporal bones was performed, and the significant regression of inflammation in both tympanic cavities as well as in paranasal sinuses was observed. There was also an improvement in hearing with the relief of tinnitus. The control pure-tone audiometry showed the curve of bone conduction in the range from 0 to 15–20 dB and the curve of the air conduction in the low and medium frequencies from 20–40 dB, and 65 at high frequencies bilaterally (Figure 4).

The left ear tympanometric curve was of A type. Normalization of inflammation parameters was also obtained (CRP 2.15; OB 9 mm). 

## 3. Discussion

Wegener's granulomatosis was described by Friedrich Wegener in 1936 [11]. It still remains a disease entity of unknown etiology, causing diagnostic difficulties. Its onset is usually nonspecific. In 1990, the American College of Rheumatology has established the criteria for confirming the diagnosis of Wegener's granulomatosis with a sensitivity of 88.2% and a specificity of 92% [12]. To recognize GW, at least two of the four criteria have to be observed. These include hematuria (more than 5 red blood cells in the visual field or the presence of the erythrocyte casts), the changes in the chest radiograph (nodules, cavities, or infiltration), ulceration of the mouth and/or nose, and positive histopathological examination [6]. In addition to the above diagnostic criteria, c-ANCA, which are positive in about 80–90% of the cases, and p-ANCA positive in approximately 10% of the cases are important [13]. These antibodies belong to a group of anti-neutrophil cytoplasmic antibodies (ANCA) damaging vascular endothelium and causing its necrosis [3].

In the initial phase of the disease, 70–95% of cases' changes demonstrate in the head and neck region [2, 5]. The lesions can be located in the nasal cavity and paranasal sinuses (60–90% of cases), in the ears (20–70% of cases and 40–70% of these cases concern the middle ear), in the larynx (5–20% of cases), and rarely in the oral cavity or parotid glands [5].

The WG lesions located in the ear mostly concern the middle ear. The ear symptoms may be the first signs of WG and may precede the primary diagnosis even a few years. The study of Bakthavachalam et al. about hearing loss in patients with WG reported that 14% of cases of hearing loss were observed before the correct diagnosis. Sensorineural hearing loss and conductive hearing loss occurred with similar frequencies, 47% and 33%, but the response for treatment was better in the group of patients with conductive hearing loss, indicating a worse prognosis in the group of patients with sensorineural hearing loss [14]. A similar study was conducted by Takagi et al. They emphasized the importance of prompt diagnosis and early implemented proper treatment for the prognosis in cases of hearing loss in the course of WG [12]. Ninety percent of cases with the middle ear involvement, one or both sides, are associated with inflammation process, causing the formation of granulation tissue in nasopharynx, leading to ulceration and stenosis of the eustachian tube and resulting in secretory otitis media [8, 15]. From twenty to forty percent of patients with SOM improve after surgery—the paracentesis with the use of prolonged ventilation drainage insertion [5]. The inner ear changes are reported in 43% of patients, causing sensorineural hearing loss. Most likely this is related to the cochlear vessels vasculitis, but the causation is not entirely clear. The pathogenesis of immune complexes or granulomatous inflammation of small vessels is suspected [5, 11]. In 24% of the WG patients, chronic otitis media with the involvement of the tympanic cavity and mastoid process is observed [8]. Dizziness is very rare. Occasionally, like in the case described herein, the ongoing disease process in the middle ear may have fulminant course, leading to bilateral facial palsy. The facial palsy may be associated with segmental nerve compression on its course in the Fallopian canal or with vasculitis [8, 15]. In the case of the facial nerve paralysis, the differential diagnosis is important and should be related to other diseases such as chronic otitis media and systemic vascular diseases including sarcoidosis or polyarteritis nodosa. Tuberculosis should also be considered [11, 15]. In 2001, Nikolaou et al. reported 22 cases of facial nerve paralysis in the course of WG [16]. At present, it is estimated that the facial nerve palsy occurs in 5% to 8% of the WG patients [8, 11, 17]. However, the bilateral facial nerve paralysis is described extremely rarely in the literature [8, 16, 17]. Preuss et al. presented a case of the bilateral facial nerve palsy, in the course of WG, with the lungs and kidneys involvement, which has been fatal due to multiple organ failure in spite of intensive treatment [18].

Surgical treatment of the Wegenr's granulomatosis patients is debatable. For many authors, it is not recommended. Described in the current literature cases of the facial nerve paralysis in a course of WG, treated surgically, shows that surgical treatment does not improve the prognosis, but could increase the risk of further facial nerve damage [10, 11, 15, 17]. However Magliulo et al. and Gottschlich et al. emphasized in their publications that in case of purulent otorrhea, not responding to antibiotics, or in case of symptoms of mastoiditis, the surgical treatment should be implemented [5, 17]. In our case, we observed rapid improvement of hearing, pain relief, and functional facial nerve recovery on the operated side, comparing to the opposite ear, suggesting the reasonability of surgical treatment.

## 4. Conclusions

Wegenr's granulomatosis with the involvement of the middle ear can proceed rapidly resulting in the form of unilateral or bilateral facial nerve palsy accompanied by mixed hearing loss. In such cases, WG should be always taken into account in the differential diagnosis. The implementation of appropriate pharmacological treatment without delay allows the hearing loss recovery and the relief of the facial nerve paralysis.

## References

[1] Szczeklik A (2006). *Choroby Wewnętrzne*.

[2] Morales-Angulo C, García-Zornoza R, Obeso-Agüera S, Calvo-Alén J, González-Gay MA (2012). Ear, nose and throat manifestations of Wegener's granulomatosis (granulomatosis with polyangiitis). *Acta Otorrinolaringológica Española*.

[3] Wierzbicka M, Puszczewicz M, Bartochowska A, Szyfter W (2012). The otologic manifestation of Wegener's granulomatosis—review of contemporary achievements in diagnostics and treatment. *Otolaryngologia Polska*.

[4] Falk RJ, Gross WL, Guillevin L (2011). Granulomatosis with polyangiitis (Wegener’s): an alternative name for Wegener’s granulomatosis. *Arthritis and Rheumatism*.

[5] Gottschlich S, Ambrosch P, Kramkowski D (2006). Head and neck manifestations of Wegener’s granulomatosis. *Rhinology*.

[6] Matuszewska A, Misterska-Skóra M, Wiland P (2010). A case of early recognized Wegener’s granulomatosis with renal involvement. Insights for early diagnosis. *Annales Academiae Medicae Stetinensis*.

[7] Barczynska T, Dankiewicz-Fares I, Bilinska-Reszkowska H, Zalewska J, Jeka S (2011). Atypical location of Wegener’s granulomatosis with breast involvement: case report. *Annales Academiae Medicae Stetinesis*.

[8] Ferri E, Armato E, Capuzzo P, Cavaleri S, Ianniello F (2007). Early diagnosis of Wegener’s granulomatosis presenting with bilateral facial paralysis and bilateral serous otitis media. *Auris Nasus Larynx*.

[9] Sadlak-Nowicka J, Łaska M, Bochniak M, Weber-Dubaniewicz M (2003). Oral aspects of Wegener’s granulomatosis. *Dental and Medical Problems*.

[10] Bibas A, Fahy C, Sneddon L, Bowdler D (2001). Facial paralysis in Wegener’s granulomatosis of the middle ear. *Journal of Laryngology and Otology*.

[11] Drinias V, Florentzson R (2004). Facial palsy and Wegener’s granulomatosis. *American Journal of Otolaryngology*.

[12] Takagi D, Nakamaru Y, Maguchi S, Furuta Y, Fukuda S (2002). Otologic manifestations of Wegener’s granulomatosis. *Laryngoscope*.

[13] Tłustochowicz W, Tłustochowicz M (2012). Systemic vasculitis. *Reumatologia*.

[14] Bakthavachalam S, Driver MS, Cox C, Spiegel JH, Grundfast KM, Merkel PA (2004). Hearing loss in Wegener’s granulomatosis. *Otology & Neurotology*.

[15] Hern JD, Hollis LJ, Mochloulis G, Montgomery PQ, Tolley NS (1996). Early diagnosis of Wegener’s granulomatosis presenting with facial nerve palsy. *Journal of Laryngology and Otology*.

[16] Nikolaou AC, Vlachtsis KC, Daniilidis MA, Petridis DG, Daniilidis IC (2001). Wegener’s granulomatosis presenting with bilateral facial nerve palsy. *European Archives of Oto-Rhino-Laryngology*.

[17] Magliulo G, Parrotto D, Alla FR, Gagliardi S (2008). Acute bilateral facial palsy and Wegener’s disease. *Otolaryngology*.

[18] Preuss SF, Stenner M, Beutner D, Laudes M, Klussmann JP (2008). Fatal course of Wegener’s granulomatosis with bilateral otomastoiditis and bilateral facial nerve palsy. *Otolaryngology*.

